# Molecular dissection of Chagas induced cardiomyopathy reveals central disease associated and druggable signaling pathways

**DOI:** 10.1371/journal.pntd.0007980

**Published:** 2020-05-20

**Authors:** Jacob M. Wozniak, Tatiana Araújo Silva, Diane Thomas, Jair L. Siqueira-Neto, James H. McKerrow, David J. Gonzalez, Claudia M. Calvet

**Affiliations:** 1 Skaggs School of Pharmacy and Pharmaceutical Sciences; University of California San Diego; La Jolla, CA, United States of America; 2 Department of Pharmacology; University of California San Diego; La Jolla, CA, United States of America; 3 Cellular Ultrastructure Laboratory; Oswaldo Cruz Institute, FIOCRUZ; Rio de Janeiro, RJ, Brazil; Instituto de Investigaciones Biotecnológicas, ARGENTINA

## Abstract

Chagas disease, the clinical presentation of *T*. *cruzi* infection, is a major human health concern. While the acute phase of Chagas disease is typically asymptomatic and self-resolving, chronically infected individuals suffer numerous sequelae later in life. Cardiomyopathies in particular are the most severe consequence of chronic Chagas disease and cannot be reversed solely by parasite load reduction. To prioritize new therapeutic targets, we unbiasedly interrogated the host signaling events in heart tissues isolated from a Chagas disease mouse model using quantitative, multiplexed proteomics. We defined the host response to infection at both the proteome and phospho-proteome levels. The proteome showed an increase in the immune response and a strong repression of several mitochondrial proteins. Complementing the proteome studies, the phospho-proteomic survey found an abundance of phospho-site alterations in plasma membrane and cytoskeletal proteins. Bioinformatic analysis of kinase activity provided substantial evidence for the activation of NDRG2 and JNK/p38 kinases during Chagas disease. A significant activation of DYRK2 and AMPKA2 and the inhibition of casein family kinases were also predicted. We concluded our analyses by linking the diseased heart proteome profile to known therapeutic interventions, uncovering a potential to target mitochondrial proteins, secreted immune effectors and core kinases for the treatment of chronic Chagas disease. Together, this study provides molecular insight into host proteome and phospho-proteome responses to *T*. *cruzi* infection in the heart for the first time, highlighting pathways that can be further validated for functional contributions to disease and suitability as drug targets.

## Author summary

Chagas disease is a significant human health concern as it can cause severe cardiomyopathies in chronically infected patients. Due to the high morbidity associated with Chagasic cardiomyopathies, it is vital to investigate new treatment options. In this study, we use state-of-the-art techniques to interrogate the host signaling events induced by chronic Chagas disease in the primary affected organ, the heart. We identify proteins and phospho-sites significantly altered upon infection, predict activated and inhibited kinases, and link our findings to known drug targets. For the first time, this study provides insight into the *in vivo* host signaling responses to *T*. *cruzi* in the heart, uncovering pathways that can be validated for contributions to disease and suitability as drug targets.

## Introduction

Chagas disease is the manifestation of an infection by the protozoan parasite *Trypanosoma cruzi*. First described in 1909 by a Brazilian physician, Carlos Chagas[1], this disease is a significant health concern, particularly in areas with low socio-economic status. Facilitated by human population flow, Chagas disease has spread out of endemic areas into more developed countries, with more than 100,000 cases in Europe[2] and 200,000–300,000 in the United States[3, 4] reported. Infected insect vectors and congenital transmission are the most common means of disease spread, accounting for up to ~96% of recorded cases (70% insect vectors, 26% congenital); blood transfusion, organ transplantation and consumption of contaminated foods also contribute to *T*. *cruzi* dissemination[5]. Historically overlooked, Chagas disease is classified as a neglected tropical disease by the World Health Organization[6] and is estimated to result in a global economic burden of > $7 billion (USD) per year[7]. Thus, Chagas disease is a major human health concern that causes significant morbidity and mortality worldwide.

The progression of Chagas disease can be classified into two phases, the acute phase and the chronic phase[8, 9]. The acute phase is asymptomatic in most cases, lasts approximately 1–2 months and usually resolves spontaneously[8]. However, if left untreated, patients can remain chronically infected, resulting in critical health concerns later in life[8]. These delayed adverse effects occur in approximately 30% of the infected individuals and include cardiac and visceral involvement, with cardiomyopathies being the most severe and frequent manifestation[8, 9]. Interstitial fibrosis of the heart is thought to be a major determinant factor for the pathogenesis of Chagas disease[5]. In fact, even after successfully lowering parasite loads with the current standard of therapy (ie. benznidazole), patients with advanced cardiomyopathies remained under high disease burden[9]. The reason for this is currently unclear, but suggestions have ranged from auto-immune responses[10, 11] to dormant, low-proliferating forms of *T*. *cruzi* that are resistant to anti-trypanosomals[12]. Regardless, directing therapies against fibrotic phenotypes of heart, in combination with trypanocidal agents, have great potential to effectively treat this disease.

Chronic Chagas disease in the heart is driven by an intense inflammatory response and excessive immune infiltration[5, 13]. Cytokines and chemokines, secreted by both cardiomyocytes[14] and invading immune cells[15], stimulate a wound-healing response (eg. extracellular matrix (ECM) deposition) from fibroblasts to repair the damaged tissue[5]. Specifically, transforming growth factor β (TGF-β)[16], tumor necrosis factor-α (TNF-α)[14], and interferon gamma (IFN-γ)[15] are central to the immune response and pathology of Chagas disease. These effectors induce a myriad of downstream signaling cascades, resulting in diverse functional outcomes from apoptosis[17] to accumulation of ECM[16, 18]. The potential intracellular signaling pathways include c-Jun N-terminal kinase (JNK)[19] and p38[20]. Recent reports from *in vitro* models of *T*. *cruzi*-host cell interaction demonstrated that cardiac fibroblasts display an increase in phosphorylation of p38 and c-Jun after infection[21]. Transcriptomic analysis showed that *T*. *cruzi* infection upregulates the JUNB gene and results in translocation of JunB to nuclei of primary human cardiomyocytes[19]. In line with this hypothesis, treatment of mice with genistein[22], a tyrosine kinase inhibitor, lowered TAK1 and JNK activity and decreased cardiac fibrosis in a hypertension model[23], suggesting this pathway is associated with cardiac remodeling. Further, SP600125[24], a canonical JNK inhibitor, is well tolerated by mice and provides protective effects for hearts in damaging conditions[25]. Despite these developments, Chagas disease progression results in alterations of other signaling pathways[20, 26] that should not be overlooked as therapeutic targets.

Conventional, single target approaches to analyze intracellular signaling pathways have been the foundation for understanding complex biology. However, these methods only focus on a few proteins and even simple stimuli are known to induce drastic molecular alterations in the intracellular milleu. Thus, systems level technologies (eg. transcriptomics and proteomics) have emerged as an effective tool to unbiasedly assess the gene expression state of various disease conditions. While total gene expression levels provide useful information, many central signaling pathways are mediated via post-translational modifications (PTMs), with minimal alterations in total protein abundance[27–29]. Therefore, proteomics has a distinct advantage relative to other -omics technologies in the ability to detect protein PTMs at a systems scale[30]. Phospho-proteomic approaches have provided a means to dissect precise signaling pathways involved in diverse biological processes, from development[31] to infectious disease[32]. A phospho-proteomic approach applied to Chagas disease models in the heart has not been attempted to our knowledge and thus has the potential to deepen the understanding of global signaling pathways affected by chronic *T*. *cruzi* infections.

In this study, we apply a phospho-proteomic workflow to interrogate chronic Chagas disease progression. This investigation constitutes a foundational examination of the global phospho-signaling response to *T*. *cruzi* in the primary affected organ, the heart. Our analyses uncover both known and previously uncharacterized alterations in total protein abundance and phosphorylation status. As expected, we captured the classical induction of IFN-mediated signaling pathways[15] and repression of mitochondrial function[33, 34]. Of significance, our unbiased approach identified new players that may have a role in disease progression including Immunity Related GTPase M (IRGM) 1 and 2 and the immune-associated, guanylate binding proteins (GBPs). In addition to total protein abundance changes, we uncovered a vast signaling network of plasma membrane and intermediate filament proteins with perturbed phosphorylation status following infection. These include new targets such as Striated Muscle Enriched Protein Kinase (SPEG), Tensin 1, BCL2 Associated Athanogene 3 (BAG3), Sorbin and SH3 domain-containing protein (SORBS) 1/2 and myosin-family proteins in addition to the previously described p38 axis. Further, we applied bioinformatics to predict active kinases, supporting the involvement of JNK and identification of new activated (DYRK2 and AMPKA2) and repressed (casein kinase family) kinases in the host response to *T*. *cruzi* infection. Finally, through the creation of a druggable disease network, we propose a number of FDA approved drugs that may be repurposed for the treatment of chagasic cardiomyopathy. Overall, this study reveals new signaling pathways modulated during chronic Chagas disease that expand the understanding of molecular mechanisms of pathogenesis and inform rational drug design.

## Results

### Mouse model of Chagas disease

**Fig 1:**
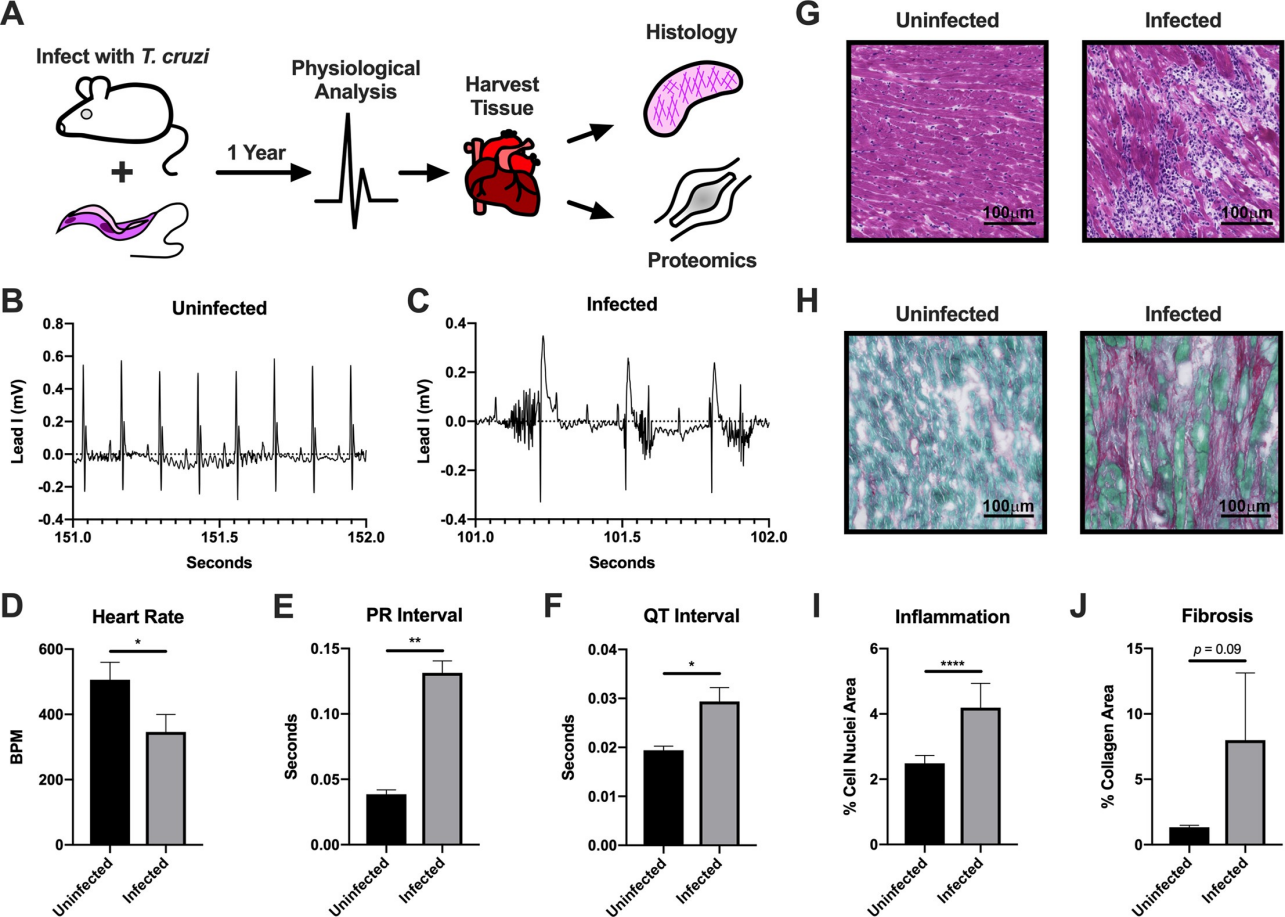
Chronic Chagas cardiomyopathy model. *A*, Schematic of model infection and analyses. *B*, Representative one-second ECG trace from uninfected mouse. *C*, Representative one-second ECG trace from infected mouse. *D*, Average heart rates from uninfected and infected animals during ECG analysis. *E*, Average PR intervals from uninfected and infected animals during ECG analysis. *F*, Average QT intervals from uninfected and infected animals during ECG analysis. *G*, Representative images of cell nuclei staining in hearts of uninfected and infected animals. Bar = 100μm. *H*, Representative images of collagen staining in hearts of uninfected and infected animals. Bar = 100μm. *I*, Quantitation of areas of cell nuclei. *J*, Quantitation of areas of collagen staining. For all graphs, significance was determined using an unpaired Student’s t-test (*p < 0.05, **p < 0.01, ****p < 0.0001).

To ensure that our infection model properly reflects Chagas disease symptoms, we performed electrocardiography and histological analyses of the infected hearts (**Fig 1A**). We found that mice infected with *T*. *cruzi* presented heart arrhythmias (**Fig 1B and 1C**), a lower heart rate (**Fig 1D**) and AV block (**Fig 1C and 1E**), which mimics human chagasic hearts[35] and are in accordance with previous mouse models of chronic *T*. *cruzi* infection using different strains of the parasite[36]. The extended QT interval observed in previous mouse infections[36] was also captured in our model (**Fig 1F**). These phenomena usually occur in parallel with interstitial fibrosis and inflammation[5]. Therefore, we performed histological analyses and confirmed that these mice displayed intense cardiac inflammation (**Fig 1G**), quantified by % area of cell nuclei (**Fig 1I**), and increased collagen deposition (red staining, **Fig 1H**) in the heart tissue. In agreement with human clinical symptoms[37], quantification of the fibrotic area demonstrated a 3-fold increase in the area occupied by collagen in cardiac sections from *T*. *cruzi* infected animals (**Fig 1J**). Together, this data validates the establishment of an *in vivo* model of chronic Chagas disease cardiomyopathy that shows clinical symptoms of heart disease similar to those observed in human patients, providing a strong foundation for subsequent analyses.

### General overview of phospho-proteomic results

**Fig 2:**
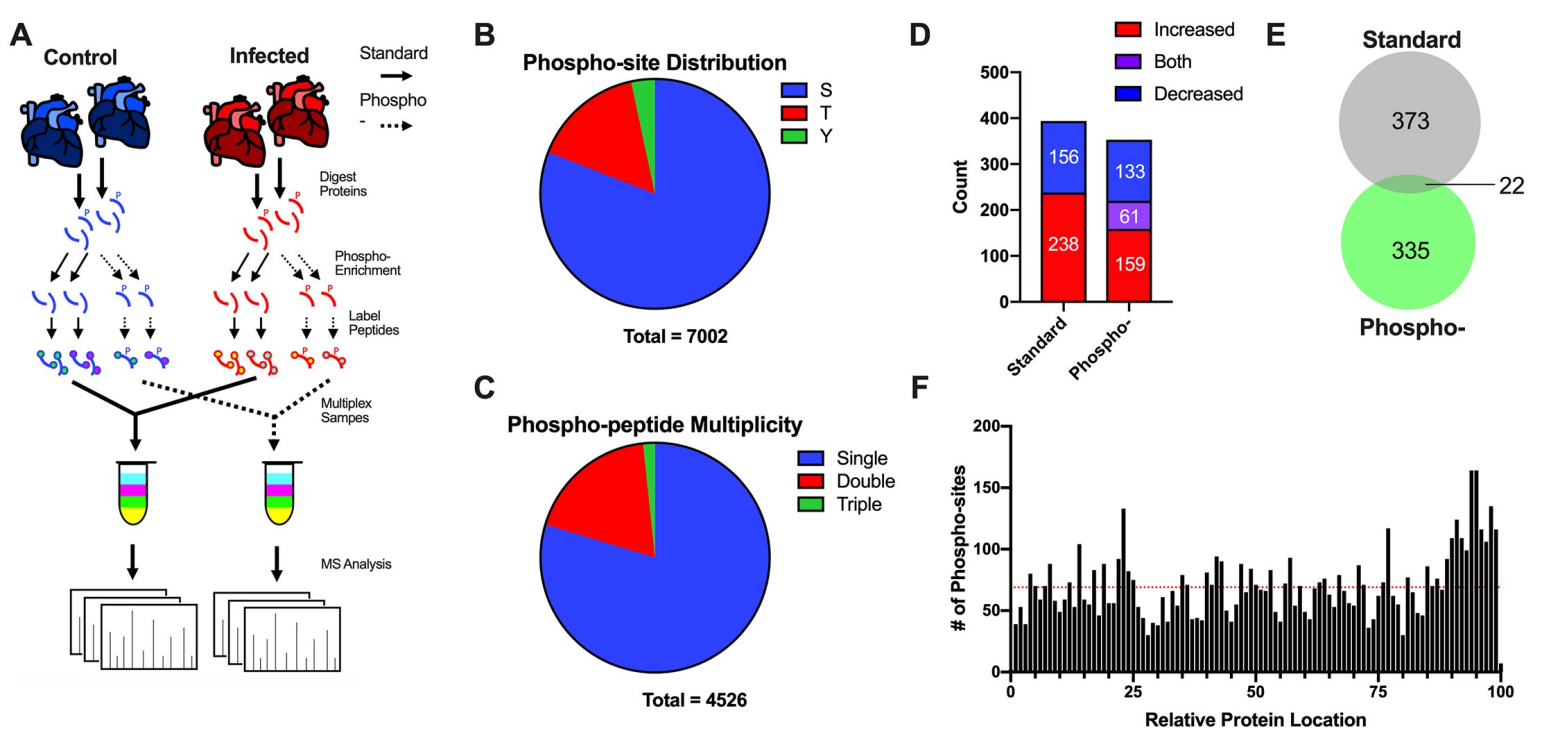
Overview of dual proteome/phospho-proteome analyses. *A*, Schematic of phospho-proteomic analysis workflow. *B*, Distribution of phospho-sites detected in this experiment. *C*, Multiplicity of phospho-peptides detected in this experiment. *D*, Numbers of significantly altered (pi score < 0.05) proteins and phospho-proteins. *E*, Venn diagram of significantly altered proteins and phospho-proteins. *F*, Relative protein locations of phospho-sites detected in this experiment.

The applied phospho-proteomic workflow (**Fig 2A**) identified and quantified 4,622 proteins (**S1 Table**) and 6,807 unique phospho-peptides at a false discovery rate (FDR) of < 1%. Our analyses revealed a high overlap of phospho-peptides that were matched to total protein abundances (**S1A Fig**). Thus, we were able to normalize 4,526 phospho-peptides to their respective total protein levels (**S2 Table**). Subsequent phospho-analyses were performed on the protein-normalized, phospho-peptide dataset. We found that the biological replicates of both the proteomic and phospho-proteomic data had low inter-replicate variation (coefficient of variation (CV) < 15% for all replicates; **S1B Fig**) and high correlation (**S1C and S1D Fig**), endorsing the reproducibility of the Chagas disease model and proteomic methods. In line with previous TiO_2_-enriched phospho-proteomic studies [38, 39], we primarily detected serine phosphorylation (81%), followed by threonine (16%) and tyrosine (3%) residues (**Fig 2B**). The majority of the detected phospho-peptides had only one phosphorylation event (80%) but a notable fraction possessed 2 or more events (20%) (**Fig 2C**). Overall, we found significant changes (pi score < 0.05)[40] in 394 proteins (238 increased, 156 decreased) and 353 phospho-proteins (133 increased, 159 decreased, 61 both) (**Fig 2D, S1E and S1F Fig**). There was low overlap between the proteins found to be significantly altered by total abundance and phosphorylation events, suggesting distinct pathway involvement (**Fig 2E**). The phospho-sites quantified in the experiments were primarily evenly distributed across the total protein length, with a slight bias for protein C-termini (**Fig 2F**). These broad analyses demonstrate high quality results and that chronic Chagas disease induces a vast rearrangement in host proteins and phospho-sites in the heart.

### Chronic Chagas disease induces an IFN-mediated immune response and represses mitochondrial proteins

**Fig 3:**
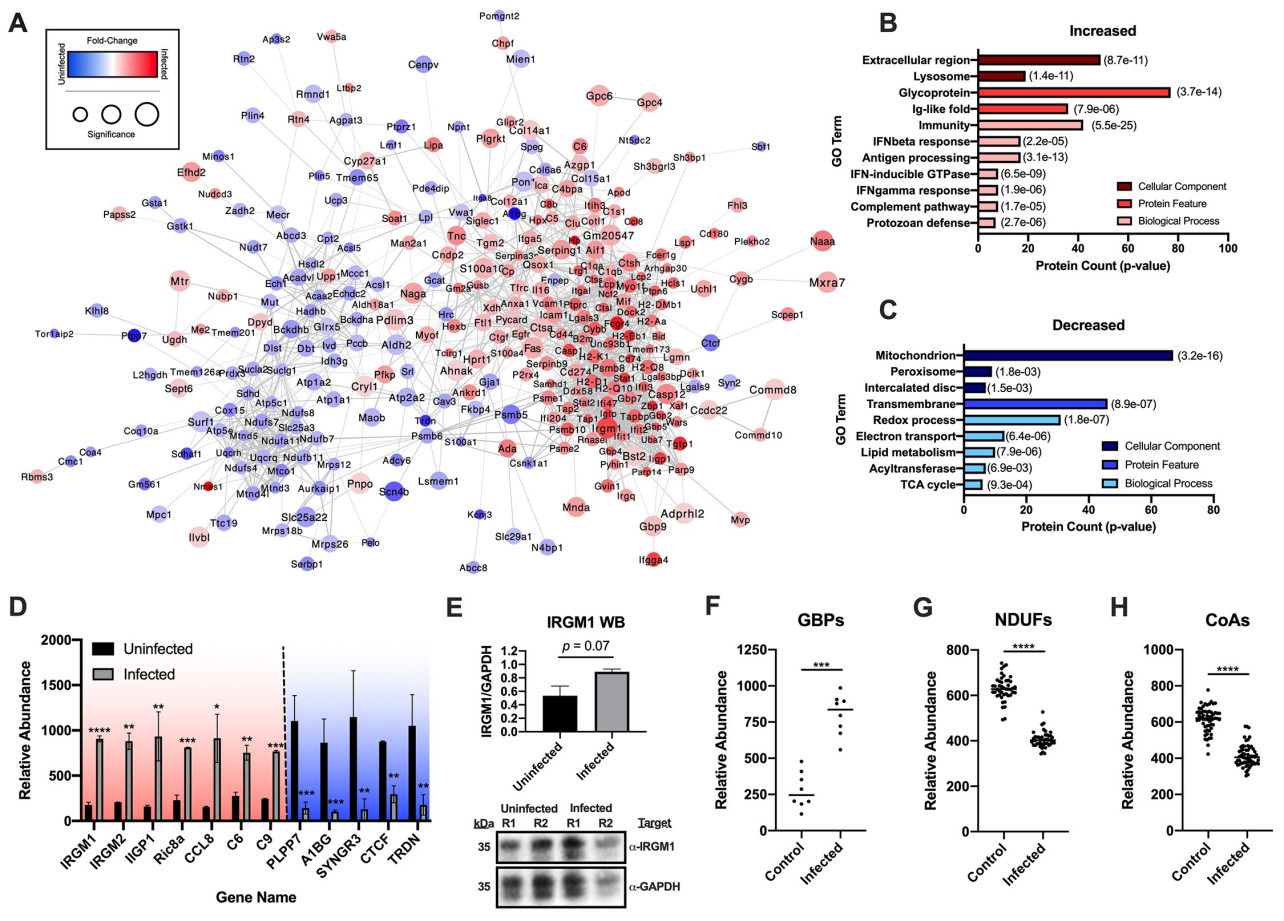
Total proteome perturbations demonstrate induction of host immune response and suppression of mitochondrial proteins. *A*, Functional protein association network of all significantly altered proteins detected in the standard proteomic workflow. *B*, GO results of significantly increased proteins. *C*, GO results of significantly decreased proteins. (Significance of enrichment is shown in parenthesis to the right of the bars). *D*, Selected increased and decreased proteins detected in the standard proteomic workflow. Significance is noted in reference to the pi score cutoffs for respective significance thresholds (* - α < 0.05; ** - α < 0.01; *** - α < 0.001; **** - α < 0.0001). *E*, Western blot validation of IRGM1 expression (R1 –replicate 1, R2 –replicate 2). *F*, Differentially expressed GBPs detected in the standard proteomic workflow. *G*, Differentially expressed NDUFs detected in the standard proteomic workflow. *H*, Differentially expressed CoA-containing enzymes detected in the standard proteomic workflow. For differentially expressed families of proteins (F-H), a paired T-test was used (*** - α < 0.001; **** - α < 0.0001).

We focused our initial analysis on the total protein abundance changes following chronic Chagas disease. We found that total levels of 394 proteins were significantly altered during infection (**Fig 2D, S1E Fig**). To gain a deeper understanding of the functional outcome of this proteome perturbation, significantly altered proteins were subject to functional protein association network and gene ontology (GO) analyses using String-DB[41] and DAVID[42, 43], respectively. We observed that the altered proteins formed a highly interconnected network consisting of two primary clusters of proteins with increasing and decreasing abundance (**Fig 3A**). Interestingly, proteins altered in the opposite directions were located within the primary clusters, suggesting that an increase of abundance of one protein may be related to the decreased abundance of another. GO analysis revealed proteins with increased abundance were primarily secreted glycoproteins involved in generating an effective immune response against protozoan invaders (**Fig 3B; S3 Table**). These changes appeared to be primarily driven by IFN-mediated signaling pathways, supporting previous studies[15]. Lysosomal proteins and antigen processing pathways were also increased (**Fig 3B**), again consistent with an IFN-driven immune response[44, 45]. In contrast, proteins with decreased abundances were primarily derived from the mitochondria (**Fig 3C**). Specifically, transmembrane proteins associated with electron transport and redox reactions were the most enriched among the decreased proteins. Peroxisomal proteins and lipid metabolic processes were also significantly reduced in the infected mice (**Fig 3C**). While it should be noted that GO analysis are not experimental and need to be validated further, the total protein level alterations during Chagas disease suggest a strong immune response and suppression of general host metabolism.

To validate the proteomic studies, we cross-examined our data against the current knowledge regarding the impact of *T*. *cruzi* infections on host cells (**S2A and S2B Fig**). It has been reported that chronic Chagas disease is associated with increased chemokine (eg. CCL2[46] and CCL5[47]) and adhesion molecule (eg. ICAM1 and VCAM1)[48] expression in the heart leading to an infiltration of pro-inflammatory immune cells. In the experiments herein, we found a significant increase in both VCAM1 and ICAM1 (**S2A Fig**) but the chemokines CCL2 or CCL5 were not identified. However, we did identify CCL8, which had an ~6-fold increase in expression (**Fig 3D**). *T*. *cruzi* infections have also been associated with increased STAT1[49], caspase[50] and MHC class I[51] expression, all of which were detected in our data (**S2A Fig**). These results suggest highly inflammatory conditions and excessive immune cell influx. To strengthen this notion, the data was mined for proteins that are primarily expressed in immune cells and should be absent in normal heart tissue to use as markers for immune invasion. We found an increase in expression of a number of immune-cell enriched markers such as: Klra2 (killer-cells), LSP1 and Fas (lymphocytes), TGTP1 (T-cells), MHC class II (antigen presenting cells) (**S2B Fig**) in our heart samples, suggesting a notable association of these cells with the heart tissue.

To put our study in the context of the current understanding of *T*. *cruzi* infections and heart disease, we performed an in-depth comparison with previous -omic studies. First, a recent genome-wide association study (GWAS) identified numerous single-nucleotide polymorphisms (SNPs) with significant relationships to Chagas cardiomyopathy in humans[52]. Comparing our list of significant proteins to the GWAS findings, we identified a number of proteins (**S2C Fig**) that were detected in the distinct experiments. We found proteins with both increased expression (SMAP2, RBP1, COL14A1 and TGM2) and decreased expression (THBD and GNG7) to have SNPs associated with Chagas-induced cardiomyopathy. The concurrent associations of SNP variation and expression level of these proteins with Chagas disease offers support for their distinct involvement in disease progression.

A survey of the literature showed that no prior proteomic studies of chagasic hearts had been reported. However, recent transcriptomic analyses have focused on 1) organ-level host responses to *T*. *cruzi* infection[53, 54] and 2) the effects of a genetically-induced cardiomyopathy[55]. These host responses may provide a means to validate the current proteomic results and be a useful comparison to our dataset. We hypothesized that a similar modulation of gene expression may be present in various organs affected by *T*. *cruzi* as well as distinct cardiomyopathy models. Indeed, we observed a notable correlation of protein levels from our study with significantly altered transcripts in both chagasic hearts (R = 0.630, p < 0.001; **S2E Fig**) and placentas (R = 0.598, *p* < 0.001; **S2F Fig**). Further, we found that the correlation of gene expression between chagasic hearts and genetically driven, dilated cardiomyopathy (R = 0.417, *p* < 0.001; **S2G Fig**) and heart failure (R = 0.425, *p* < 0.001; **S2H Fig**) was also significant, albeit with lower correlation values. Strikingly, 80–90% of the significant genes identified in both studies were altered in the same direction (**S2E–S2H Fig**), suggesting there are similar mechanisms involved in these distinct models. As expected, this proportion was highest for proteins (this study) and transcripts from chagasic hearts (>96% agreement; **S2E Fig**). Together, the above analyses demonstrate the concordance of our results with previous studies, supporting the validity of our findings.

In addition to previously known associations to *T*. *cruzi* infections, our unbiased, systems-level assessment found a number of proteins that have not yet been interrogated in the context of Chagas disease. Interesting proteins were ranked by pi-score, a metric that combines both fold-change and *p*-value. For example, we found additional, immune-related proteins increased in response to *T*. *cruzi* infection including: IRGM1/2, IIGP1, CCL8, C6, C9 (**Fig 3D and 3E**), among others. We also noted an increase in all of the guanylate-binding proteins (GBPs) detected in our analysis (**Fig 3F**). On the other hand, we detected a decrease in abundance of proteins with no prior association to Chagas disease or immune responses. The most significantly altered among these include: PLPP7, A1BG, SYNGR3, CTCF and TRDN (**Fig 3D**). As mentioned above, our GO pathway analysis highlighted a decrease in mitochondrial proteins related to redox and electron transport (**Fig 3C**). Specifically, we noted a decrease in nearly all of the NADH dehydrogenases (NDUFs) (**Fig 3G**) and coenzyme A-containing proteins (**Fig 3H**) detected in our experiments. These findings are in-line with previous reports of decreased mitochondrial function[33] and identify the major protein families affected. These analyses demonstrate that chronic Chagas disease drives an IFN-mediated immune response and suppresses mitochondrial pathways in murine hearts. Our global proteomic data are substantiated by comparisons with previous studies and western blot validation. These results also highlight new proteins that may be involved in Chagas disease progression.

### The phospho-proteome of chagasic hearts

**Fig 4:**
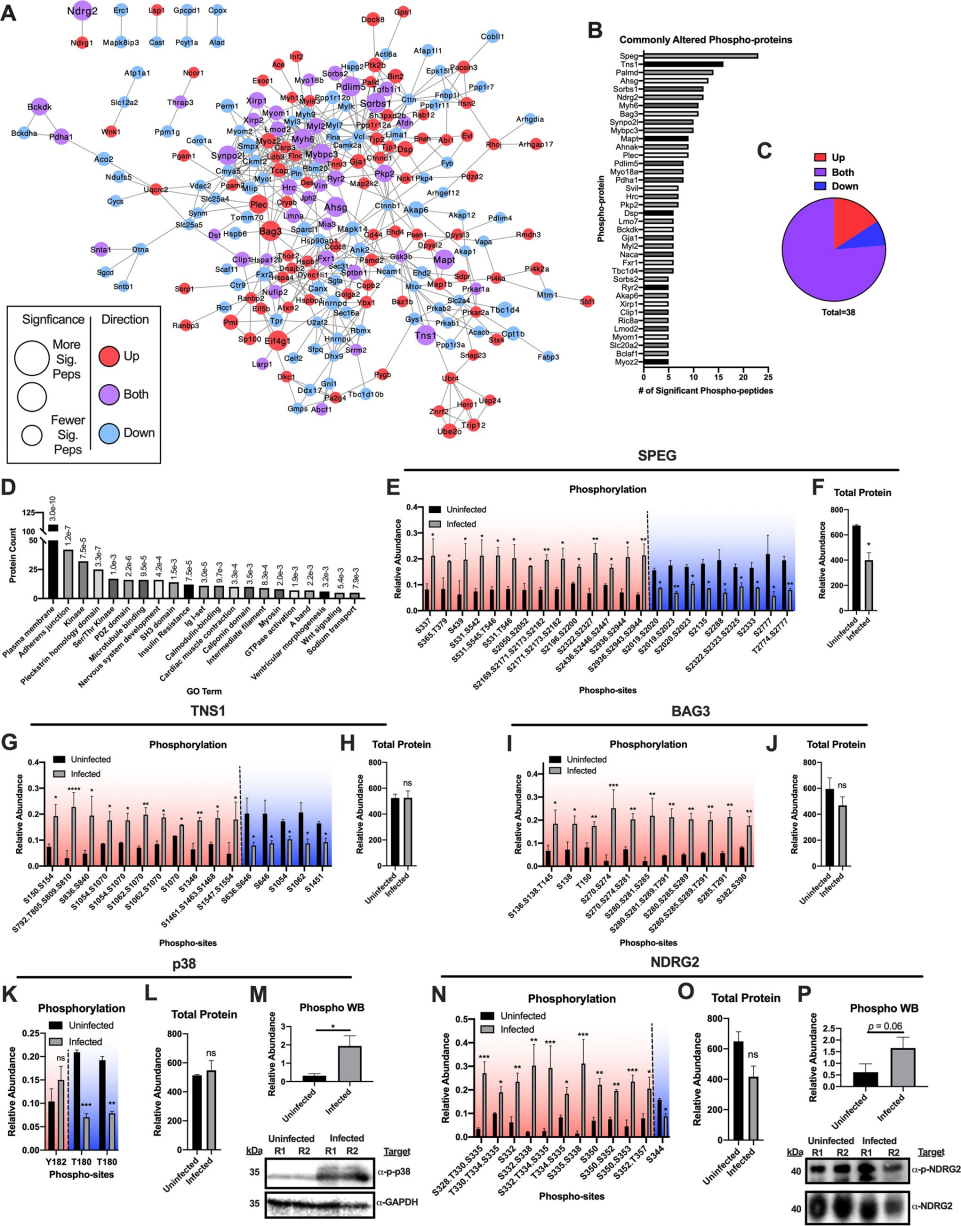
Phospho-proteome interrogation reveals alterations in membrane and cytoskeletal protein phosphorylation and activation of p38. *A*, Functional protein association network of all proteins with significantly altered phospho-peptides. *B*, Bar graph of proteins with 5 or more significantly altered phospho-peptides. *C*, Pie chart depicting the phospho-peptide direction shifts of proteins with 5 or more significantly altered phospho-peptides *D*, GO results of all proteins with significantly altered phospho-peptides. Graph depicts the number of phospho-proteins in the respective ontology with enrichment *p*-values displayed above the bar. *E*, Bar graphs of significantly altered SPEG phospho-peptides. *F*, Bar graph of total SPEG protein. *G*, Bar graphs of significantly altered TNS1 phospho-peptides. *H*, Bar graph of total TNS1 protein. *I*, Bar graphs of significantly altered BAG3 phospho-peptides. *J*, Bar graph of total BAG3 protein. *K*, Bar graphs of significantly altered p38 phospho-peptides. *L*, Bar graph of total p38 protein. *M*, Western blot validation of p38 phosphorylation status. *N*, Bar graphs of significantly altered NDRG2 phospho-peptides. *O*, Bar graph of total NDRG2 protein. *P*, Western blot validation of NDRG2 phosphorylation status. For western blot figures, R1 –replicate 1 and R2 –replicate 2. Significance is noted in reference to the pi score cutoffs for respective significance thresholds (* - α < 0.05; ** - α < 0.01; *** - α < 0.001; **** - α < 0.0001).

While total protein abundances provide useful information, many cellular signaling pathways are mediated via phosphorylation, with minimal alterations in total abundance. Therefore, we employed a phospho-proteomic approach to dissect signaling pathways affected by chronic Chagas disease. We found that phospho-sites on 353 phospho-proteins were significantly altered following infection. The majority (83%) of these phospho-proteins had only increased (159) or decreased (133) phosphorylation, but a subset had both increased and decreased phospho-sites (61) (**Fig 2D**). Creating a functional protein association network[41] (**Fig 4A**), we observed that proteins with opposite directions of phosphorylation abundance were highly interconnected, indicating that the increase of phosphorylation of one protein is associated with a decrease in phosphorylation of related proteins, and vice-versa. While the majority of phospho-proteins possessed only one significantly altered phospho-peptide, ~40 proteins had five or more significantly altered phospho-peptides (**Fig 4B**). Interestingly, most of these proteins had both increasing and decreasing phosphorylation sites (**Fig 4C**) and were centrally located in our network, putting forth their roles as key signaling hubs in Chagas-induced cardiomyopathy.

### Chronic Chagas disease alters membrane protein and microtubule phosphorylation

To determine the global signaling pathways altered during chronic Chagas disease, we performed GO analysis on all of the phospho-proteins with significantly altered phospho-sites (**Fig 4D; S4 Table**). We found highly significant enrichments for proteins localized to the plasma membrane, adherens junctions and intermediate filaments. As expected, we noted significant enrichments for many proteins involved in intracellular signaling such as kinases, calmodulin-binding proteins, and GTPases. Protein domains that govern signaling pathways from the plasma membrane through the cytoskeleton, such as pleckstrin-homology, PDZ and SH3 domains, were also enriched in this dataset. These results indicate that chronic Chagas disease has the most significant effects on intracellular signaling pathways bridging the plasma membrane and the cytoskeleton.

Specific proteins of interest were prioritized based on 1) the number and significance of altered phospho-sites, 2) their involvement in plasma membrane and cytoskeletal signaling, and 3) their relationship to previously described Chagas disease processes or other cell stresses. These include: striated muscle preferentially expressed protein kinase (SPEG), Tensin 1, BCL2 Associated Athanogene 3 (BAG3), Sorbin and SH3 domain-containing protein (SORBS) 1/2 and a number of myosin-related proteins (MYH6, MYBPC3, MYO18A, MYL2, MYOM1, MYOZ2). SPEG had the highest number of significantly altered phospho-peptides (23 phospho-peptides; **Fig 4B, 4E and 4F**) followed by Tensin 1 (16 phospho-peptides; **Fig 4B, 4G and 4H**). Both of these proteins had phospho-sites that increased or decreased in abundance, suggesting distinct kinases/phosphatase regulation of these proteins. BAG3 contained 11 significantly altered phospho-peptides that were all increased (**Fig 4B, 4I and 4J**). SORBS1/2 (**S3A–S3D Fig**) and a number of myosin proteins (**S3E–S3P Fig**) also had multiple, significant phosphorylation shifts in response to *T*. *cruzi* infection. The fact that we captured multiple members of these protein families argues for their importance in the host response to *T*. *cruzi*.

In accordance with previous *in vitro* studies with cardiac fibroblasts[21] and colon cells[20], we detected an increase in p38 Y182 phosphorylation following chronic *T*. *cruzi* infection in mice (**Fig 4K–4M**). Interestingly, we also detected a significant decrease in singly phosphorylated p38 T180 peptides **(Fig 4K**). Residues T180 and Y182 of p38 are sequentially phosphorylated in response to pro-inflammatory conditions[56]. The observed decrease in singly phosphorylated p38 T180 peptides may be a result of an increase in the doubly phosphorylated species, which was not detected in our phospho-proteomic data. To clarify these findings, we performed western blot of p38 Y182 (**Fig 4M**), confirming its increased phosphorylation status. We also detected increased phosphorylation of N-Myc Downstream-Regulated Gene 2 (NDRG2) (**Fig 4N–4P**), which can act upstream of p38[57, 58]. These results further support the activation of this pathway in the host response to infection. Overall, the above analyses highlight global pathways and specific proteins whose phosphorylation status is modulated during chronic Chagas disease.

### Prediction of kinase activity during chronic Chagas disease reveals increased JNK/DYRK and decreased CK2 activity

Detecting alterations in phospho-site abundances is crucial to understanding the ultimate outcomes of various signaling processes. However, linking phospho-site abundance to specific kinases provides more mechanistic insight into the underlying biology and can identify potential drug targets. Therefore, in an effort to identify kinase-substrate interactions with increased and decreased activity during Chagas disease progression, we undertook a two-pronged approach.

**Fig 5:**
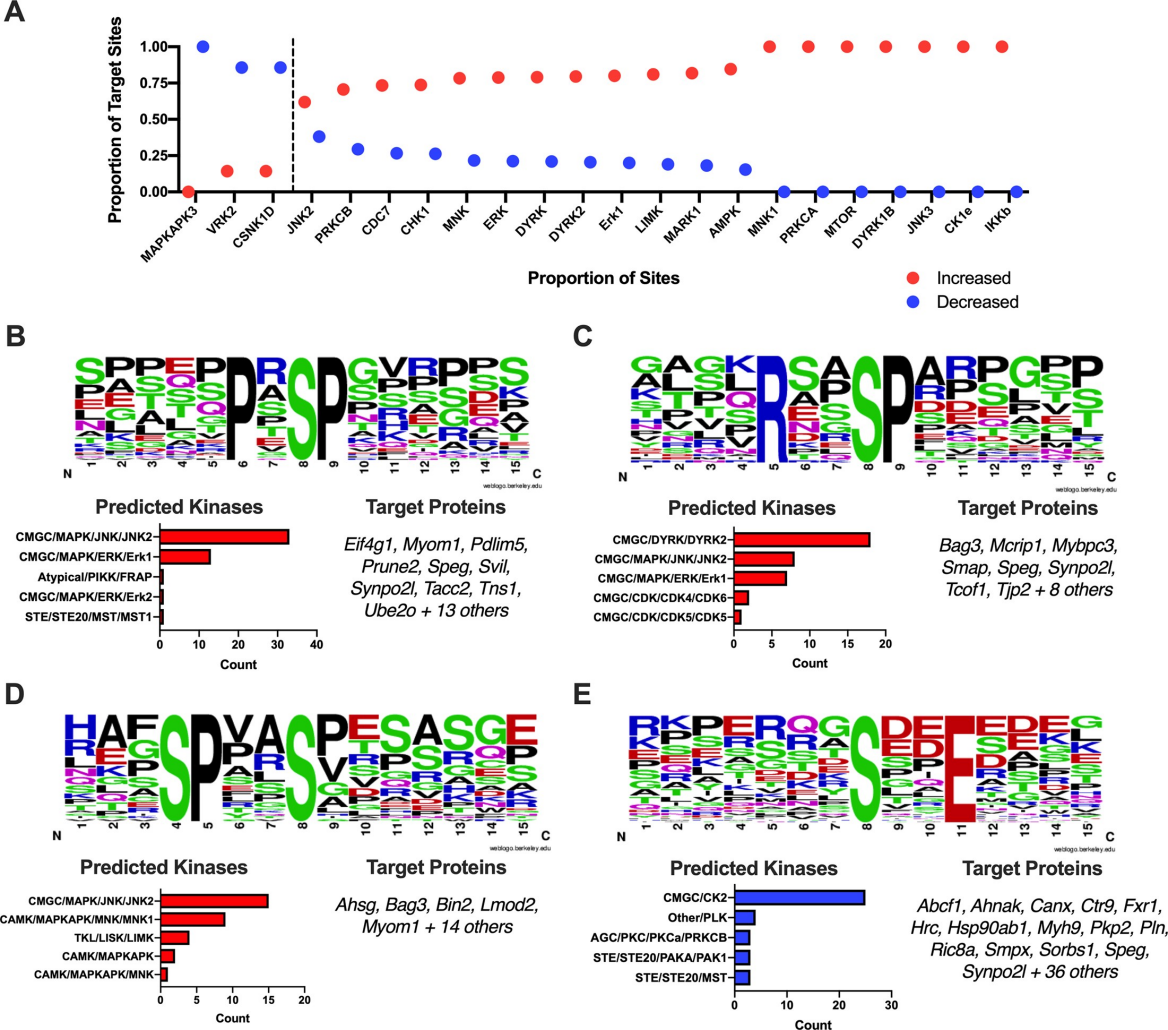
Bioinformatic kinase prediction uncovers stimulation of JNK and DYRK2 and suppression CK2 activities. *A*, Kinases with significant proportions (Chi-squared p-value < 0.05) of differentially expressed phospho-sites. *B*, Logo of enriched motif “PxS_p_P” (top) with bar graph of the number of predicted kinase sites (bottom left) and list of target proteins (bottom right). *C*, Logo of enriched motif “RxxS_p_P” (top) with bar graph of the number of predicted kinase sites (bottom left) and list of target proteins (bottom right). *D*, Logo of enriched motif “SPxxS_p_” (top) with bar graph of the number of predicted kinase sites (bottom left) and list of target proteins (bottom right). *E*, Logo of enriched motif “S_p_xxE” (top) with bar graph of the number of predicted kinase sites (bottom left) and list of target proteins (bottom right).

First, we used the group-based prediction (GPS) tool[59] to predict the kinases that target the significant phosphorylation sites detected in our experiments. This analysis predicted substrates for 125 kinases within our dataset (**S5 Table**), with AGC-group kinases having the most unique members (25 kinases; **S4A Fig**) and CMGC-group kinases targeting the most sites (422 sites; **S4B Fig**). We then compared which kinases were predicted for phosphorylation sites with increased vs. decreased abundance (**Fig 5A; S4C–S4F Fig**). We found that more site-kinase pairs were predicted to have increased activity in the infected samples than in the controls (**S4C Fig**). Stratifying predicted kinases by kinase groups and families suggests that there is a significant increase in activity from the CAMK, CMGC and TKL groups (**S4D Fig**) and MAPK, PKC, MAPKAPK, DYRK and LISK families (**S4E Fig**). Individual kinases with increased activity include: IKKB, CDC28, MNK1, AMPKA2, MARK1, LIMK, ERK1, MAPK2K2, JNK2 and DYRK2 (**Fig 5A, S4F Fig**). In contrast, the kinases with the most decreased activity are VRK2, CSNK1D, TTBK1 and ROCK. Interestingly, we identified kinases that are predicted to target phospho-sites with both increased and decreased abundance, such as FRAP, PIM1, PAK1 and RAF, suggesting that the specificity of these kinases may be modulated as opposed to overall activity.

Second, we extracted the flanking sequences of each phospho-site using the *PTMphinder* R package[60] and used the motif-x algorithm[61, 62] to identify enriched peptide motifs that were either increased or decreased during infection (**S6 Table**). Through this analysis we found that phospho-sites within the motifs: PxS_p_P (**Fig 5B**), RxxS_p_P (**Fig 5C**) and SPxxS_p_ (**Fig 5D**) were increased following infection and phospho-sites within the S_p_xxE motif (**Fig 5E**) were decreased following infection. We then linked the phospho-sites within these motifs to their predicted kinases from the GPS analysis to create putative kinase-substrate interactions (**Fig 5B–5E; S7 Table**). This dual prediction approach provides further evidence for an activation of JNK/p38 family kinases and downstream MNK kinases, but also uncovers unexpected kinases such as DYRK2 (increased activity) and CK2 (decreased activity). Overall, both known (eg. JNK) and previously unknown (eg. DYRK2, CK2) kinases were predicted to be differentially activated in response to *T*. *cruzi* infection. We also identified their respective substrates for further validation as functional players in Chagas disease progression.

### The druggable network of chagasic hearts

**Fig 6:**
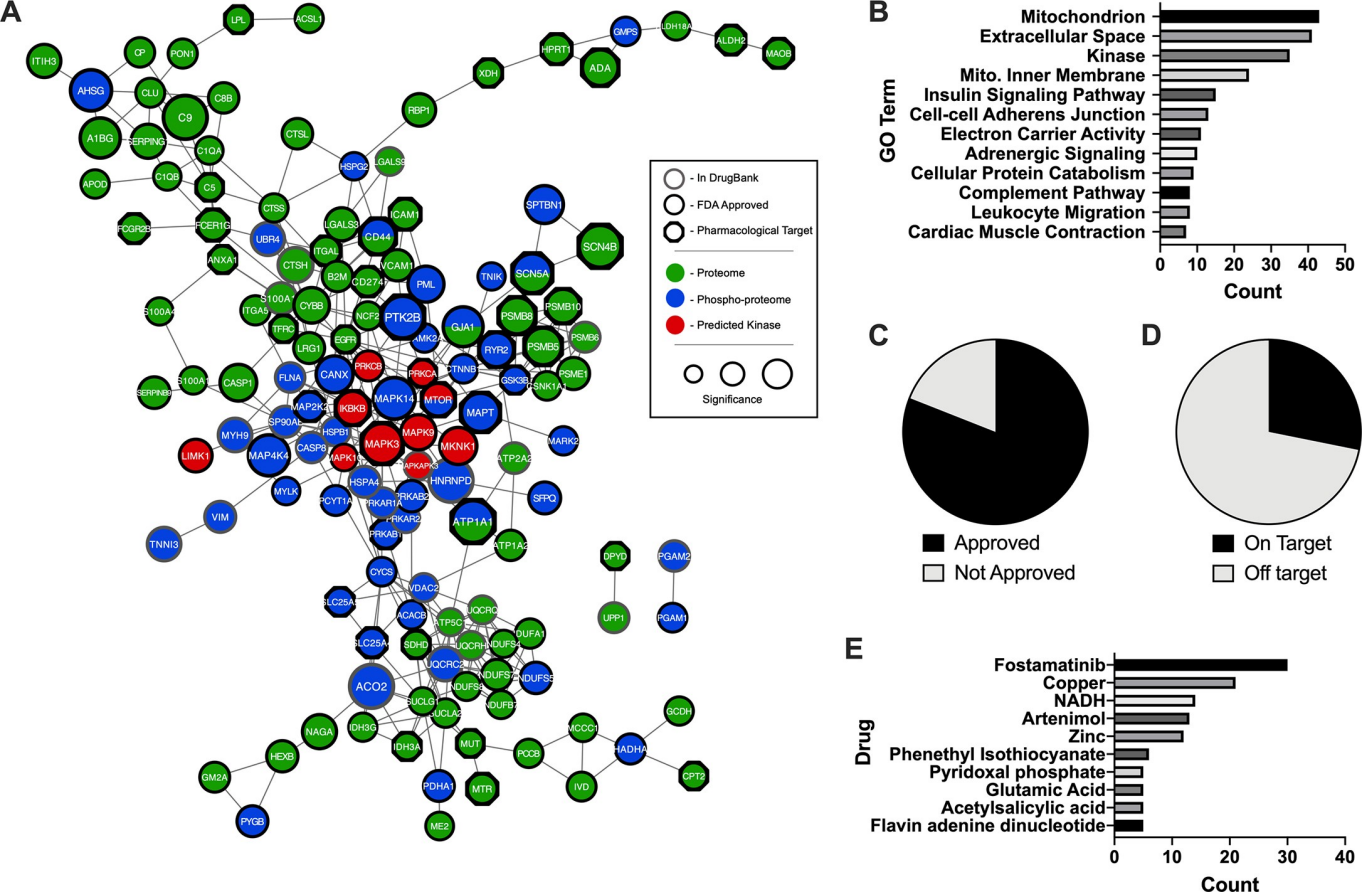
Druggable network of chagasic hearts. *A*, Functional protein association network of significantly altered proteins, phospho-proteins and predicted kinases that are known interactors of drugs from DrugBank. *B*, GO analysis of proteins in the druggable network. Pie charts of the *C*, approval status and *D*, intended target status of drugs that interact with proteins in the druggable network. *E*, Bar chart of drugs with 5 or more targets in the druggable network.

To elucidate druggable signaling pathways that can be further explored for their therapeutic potential, we linked the significant proteomic, phospho-proteomic and kinase prediction results to known drug targets within the DrugBank database[63, 64] (**S8 Table**). Visualizing these druggable proteins using functional protein association analysis, we found that they formed a highly interconnected network with the predicted kinases at the center, closely followed by phospho-proteins, and the standard proteins residing in the distal regions (**Fig 6A**). Gene ontology analysis revealed that the most druggable pathways are mitochondria, secreted proteins, kinases and cell-cell adherens junctions (**Fig 6B; S9 Table**), all of which are intimately linked to *T*. *cruzi* infections and heart disease. Strikingly, more than 80% of the proteins in our network are known interactors with FDA approved drugs (**Fig 6C**); approximately 35% of which are linked to the intended, on-target effects (**Fig 6D**). To identify drugs that could broadly impact multiple proteins altered during chronic *T*. *cruzi* infections, putative therapeutics were ranked based on the total number of targets present in the network (**Fig 6E**). We found that Fostamatinib, Artenimol, metals bound by secreted immune proteins (zinc and copper) and NADH may have a strong influence on the outcomes of *T*. *cruzi* infections. Of note, acetylsalicylic acid (aspirin), emerged as one of the most influential drugs in our network, and has been demonstrated to be beneficial for the host during *T*. *cruzi* infections [65, 66], specifically at later stages of the disease[65].

## Discussion

Cardiomyopathy is the most common and severe pathological result of chronic Chagas disease[5, 8, 13]. While studies focusing on individual host factors have provided insight into specific mechanisms of pathogenesis, proteins typically work in co-regulated networks with significant involvement of PTMs, which are often overlooked in standard assays. Therefore, a systems-level analysis would represent a significant step toward understanding the signaling pathways and molecular mechanisms underlying disease progression. Previous studies applying proteomics to investigate Chagas disease have mostly been focused on serum, looking for disease progression markers using two-dimensional gel electrophoresis prior to mass spectrometry in rats[67] and human patients[68, 69]. Also, proteomic and phospho-proteomic approaches have been targeted toward the parasite itself, describing its response to nutritional stress[70] and proteins modulated during the differentiation process[71, 72]. However, a large-scale, phospho-proteomic analysis of chagasic hearts, one of the most affected organs, has not been performed. To address this gap in knowledge, we performed a multiplexed, quantitative phospho-proteomic analysis of an *in vivo* murine model of Chagas-induced cardiomyopathy. We find that our model possesses common clinical symptoms of chagasic hearts[35, 36], indicating that it accurately reflects human biology. While this manuscript describes findings based on two, independent, biological replicates, we believe that the agreement in our replicates (S1 Fig), particularly in the proteins/phospho-sites displayed in the figures, and comparisons to previous studies (S3 Fig), largely substantiate the dataset. However, further analysis is necessary to biologically confirm the targets appointed through our study.

We compared our proteomic expression results to previously published studies including conventional assays (eg. western blot and histology) as well as GWAS and transcriptome analyses. Together this helped validate our proteomic and prioritize interesting proteins for analysis. From this meta-analysis, we found that the host response to *T*. *cruzi* is significantly correlated across distinct organs and that chagasic hearts follow gene expression profiles similar to other cardiomyopathies. We also found a number of proteins that were differentially expressed in our proteomic data and possessed SNP-associations to Chagas disease progression. Notable among these is retinol-binding protein 1 (RBP1), which was significantly increased upon infection and linked to Chagas cardiomyopathy through GWAS studies[52]. Rbp1 functions in the uptake and storage of retinol (vitamin A)[73], a molecule with anti-oxidant[74] and immune-related[75] activities. Further, isotretinoin, a naturally-occurring derivative of retinol[76] has been shown to have promising trypanocidal effects in the nanomolar concentration range[77]. Due to the high structural similarity of retinol and isotretinoin, it seems plausible that increased Rbp1 could also increase intracellular isotretinoin concentrations, acting as a host defense mechanism against *T*. *cruzi* or chronic inflammation. Treatment of *T*. *cruzi* infections with vitamin C has been shown to have myocardial protective effects[78] and retinol can help defend against many forms of cardiovascular disease[74], however, treatment of *T*. *cruzi* infections with either retinol or isotretinoin has not yet been tested.

Other interesting groups of proteins include immune-related proteins, with both described and previously undescribed associations with Chagas disease. For example, we verified previous reports of an upregulation of the adhesion molecules VCAM1 and ICAM1[48] and the chemokine CCL8[54] which is thought to enable excessive immune infiltration. Previous studies identified CCL5 as a primary contributor to immune influx[47]. Both CCL5[79] and CCL8[80] can interact with CCR1, CCR3 and CCR5 on target cells inducing immune recruitment to the site of inflammation. Their co-expression increase suggests a redundant mechanism in the chemotaxis and could result in excessive immune infiltration.

A striking finding in our standard proteomic data was the increase in nearly the entire family of guanylate binding proteins (GBPs). Previous studies have indicated an increase of expression of GBP2 and GBP6 following *T*. *cruzi* infection in both the heart and the placenta[53, 54], but not in response to genetic cardiomyopathies[39]. However, our findings include the additional family members: GBP2B, GBP4, GBP5, GBP7 and GBP9. GBPs are induced by IFN-γ as a host defense against invading pathogens[81, 82] and are thought to function as a complex, supporting the precise co-regulation observed in the present study. Of note, GBP1, along with GBP2, 4 and 5, has been shown to localize to *T*. *gondii* containing vacuoles[83]. Further, it has been shown that mice deficient in GBP2 are highly susceptible to *T*. *gondii* infections[84]. While GBP1 was demonstrated to not co-localize with *T*. *cruzi*[83], the alternate GBPs detected in our experiments might be involved in host recognition of *T*. *cruzi*. This data demonstrates a mechanism of host response to *T*. *cruzi* that is conserved in multiple organs and highlights the additional proteins that are co-regulated in the heart. These results warrant further investigation into which specific GBPs functionally respond to *T*. *cruzi* and the ultimate consequences of this interaction.

In contrast to the proteins with increased abundance mentioned above, we detected a strong signature of mitochondrial proteins being significantly downregulated following infection. The dysfunction in mitochondria following *T*. *cruzi* infections is well known[33], but poorly understood. Previous studies have exhibited *T*. *cruzi* co-localizing with mitochondria via their single flagella[85]. Mitochondrial dysfunction is described to start in the acute phase of the infection, continuing to a reduction of oxidative phosphorylation capacity in the heart of mice during chronic infection[86]. In addition, a decline in the activities of the respiratory complex III and antioxidant enzymes (MnSOD and GPX) as well as in GSH contents has been observed in chronic Chagas patients[87]. With respect to chronic *T*. *cruzi* mouse infections, the current study, supported in part by previous transcriptome analyses[54], have demonstrated a decrease in many mitochondrial proteins. Our analyses have identified NDUFs and CoA-containing enzymes as primary contributors to this observed mitochondrial signature. NDUFs and CoA-containing enzymes are involved in many metabolic functions including electron transport and fatty acid synthesis. Intriguingly, it has been demonstrated that *T*. *cruzi* actively scavenges host long-chain fatty acids to promote its intracellular survival, a mechanism that was further validated by showing reduced parasite proliferation in fibroblasts lacking *de novo* triacylglycerol biosynthesis[88]. The downregulation of NDUFs and CoA enzymes in the heart may reflect a host effort to effectively starve out the parasite. Unfortunately, this decrease in mitochondrial function can also contribute to cardiomyopathy progression[89]. By identifying the specific proteins involved, it may be possible to target therapeutic approaches that enhance myocyte function and survival while simultaneously starving the parasite. As such, our studies reinforce previously described interplays of host-parasite lipid metabolism and highlight specific host proteins likely to be involved.

In addition to total protein expression, our analysis captured the first shotgun, phospho-proteomic analysis of the interaction of *T*. *cruzi* with host cells *in vivo*. We found drastic alterations in host phosphorylation status following infection as evidenced by significant changes of phospho-site abundance in 353 unique phospho-proteins. Functional protein association analysis[41] highlighted potential key players, which sat central to the network with large numbers of phosphorylation changes (up to 23 distinct phospho-peptides significantly altered). Specifically, SPEG, Tensin 1, BAG3, SORBS1/2 and a number of myosin-related proteins (MYH6, MYBPC3, MYO18A, MYL2, MYOM1, MYOZ2) emerged as integral components of this network. Of note, all of these proteins have been linked to proper heart function and/or implicated in the development of cardiomyopathies[90–99]. Thus, their differential phosphorylation in response to *T*. *cruzi* may be mechanistically related to the development of severe disease states.

Chronic Chagas disease drives severe inflammation and fibrosis in the heart, and our phospho-proteomic analyses found substantial evidence that the primary implicated intracellular signaling pathways are c-Jun N-terminal kinase (JNK) and p38. First, we found elevated phosphorylation of p38 at Y182, a known activation site. Second, we detected an increase in phosphorylation of NDRG2, a cell-stress related protein. Phosphorylation of p38 has been described for *in vitro* models of *T*. *cruzi* infection in colon cells (18) and cardiac fibroblasts[21]. p38 was also found increased during *T*. *cruzi* acute infection[100], but our data shows for the first time that p38 might be implicated in chronic Chagas cardiomyopathy in a model that displays clinical symptoms of heart failure. Overexpression of NDRG2 has been shown to increase p38 phosphorylation[58], suggesting it can function upstream. The observed increase in phosphorylation of NDRG2 provides further evidence for activation of this pathway. Finally, our dual kinase-substrate prediction analysis reported an increase in JNK activity, particularly at phospho-sites within the motifs PxS_p_P and SPxxS_p_. Of note, many of the highly altered phospho-proteins of interest mentioned above possess predicted JNK target sequences, suggesting downstream involvement in this signaling pathway. Together, this data supports a central role of JNK and p38 signaling pathways in Chagas disease progression and identifies new components both up- and downstream.

NDRG2 also targets other signaling pathways. Reports from cancer studies demonstrated that NDRG2 inhibits c-Myc expression by suppressing the expression of β-catenin[101] and reduces c-Jun phosphorylation and cyclin D1 expression resulting in suppression of cell proliferation in human colon carcinoma cells[102]. NDRG2 can also activate glycogen synthase kinase 3β (GSK-3β), impacting β-catenin signaling[103] and potentially contributing to cardiomyopathy establishment as the GSK3 family has been implicated in the regulation of cardiac remodeling downstream of the PI3K–Akt pathway[104]. NDRG2 has been described to be involved in survival-associated proteins and pathways with an anti-apoptotic effect, repressing the apoptotic activator Bcl-2-associated X protein (BAX)[105]. In addition, NDRG2 expression inhibits signal transducer and activator of transcription 3 (STAT3) activation[106], which can result in dysregulation of the mitochondria[107] and possibly be involved in the mitochondrial dysfunction observed in *T*. *cruzi* infection. Finally, NDRG2 has been implicated with the stress response in cardiac ischemia/reperfusion[108]. Altogether, NDRG2 constitutes a new therapeutic target for treatment of Chagas chronic cardiomyopathy.

In addition to activation of JNK and p38 pathways above, our analysis predicted an activation of IKKB, MNK1 and DYRK2 and suppression of CK2, CSNK1D and VRK2. Activation of IKKB and MNK1 is logical given the highly inflammatory conditions and activation of JNK/p38 described above[109, 110]. IKKB phosphorylates the NFKB Inhibitor Alpha in the NFKB complex, causing dissociation of the inhibitor and activation of NFKB in response to pro-inflammatory stimuli[111]. MNK1 is a MAPK activated protein (MAPKAP) and functions downstream of p38[112]. Upon activation, MNK1 phosphorylates downstream substrates, primarily involved in translational regulation, including eIF4E and eIF4G[112, 113]. While we didn’t detect any spectra assigned to eIF4E, we detected significant increases in phosphorylation of eIF4G at both S1231 and S1238, further reinforcing activation of this pathway. DYRK2 functions to regulate cell cycle and proliferation[114], apoptosis[115] and organization of the cytoskeleton[116]. We predicted DYRK2 to phosphorylate a number of interesting proteins from our phospho-hits including SPEG, BAG3, NDRG2, MYBPC3 and MYO18A. The potential for DYRK2 to phosphorylate these central proteins and also affect cytoskeletal organization make it an interesting finding for further interrogation. Finally, we noted an intriguing downregulation of CK2, CSNK1D and VRK2 activity, all kinases related to casein kinase families. VRK2 has been previously shown to downregulate JNK activity[117, 118]. Further, CSNK1D has been hypothesized to be phosphorylated by p38 at a regulatory site, reducing its activity[119]. Thus, the inactivation of these kinases further supports JNK and p38 activation in our model. On the other hand, there are conflicting reports on the impact of CK2 on JNK activity in response to inflammation[120–123]. It is important to note that, while CK2 activity was predicted to decrease, this finding was primarily linked to sites within S_p_xxE motifs. A nearly equivalent number of other CK2-predicted sites were found to be increased; however, they showed no bias toward the S_p_xxE motif. These results demonstrate a decrease in CK2 activity against specific sites, suggesting the specificity of CK2 may be altered rather than a global decrease in activity.

We conclude our study by identifying FDA approved drugs that could be repurposed for the treatment of Chagas disease in the chronic phase. This analysis indicates that therapeutic targeting of the central kinases highlighted herein (such as JNK, p38 and IKKB) could be an effective means to influence a variety of downstream proteomic and phospho-proteomic alterations. Fostamatinib, a kinase inhibitor recently approved for rheumatoid arthritis and immune thrombocytopenic purpura[124], has affinity for all these targets and thus represents a means to simultaneously disrupt these key signaling pathways. On the other hand, targeting a particular group of downstream effectors (eg. complement proteins, NDUFs, and the proteasome) could be implemented as a more focused therapy. For example, zinc supplementation has been shown to reduce complement activity in age-related macular degeneration[125]. Effectively blocking complement deposition could reduce the disease burden associated with high inflammatory conditions of the chagasic heart. Interestingly, we identified a large number of antibody-based, anti-inflammatory therapeutics (Efalizubmab, Natalizumab, etc.) that also interact with complement proteins (C1QA and C1QB), as well as EGFR and immune adhesion molecules (ICAM1, ITGAL). These targeted therapies may be useful in reducing the inflammatory conditions of the heart, particularly when used in combination with zinc or other immuno-modulatory agents. As with any immune suppressing drugs, there is an increased risk of infection for patients taking the medication[126]. Further, excessive zinc can also lead to immune dysfunction[127]. Determining which approach would be most effective at reducing disease burden, while simultaneously preserving normal cardiac function and immune system health, would represent a critical step toward developing new therapeutic options for Chagas cardiomyopathies. A thorough assessment of the impact of the identified pharmacological agents on disease progression should be pursued to build further evidence for the roles of these proteins and tease out precise mechanisms of action.

Overall, this study presents a comprehensive view of the molecular underpinnings of chagasic hearts. We identify specific host pathways and proteins that respond to *T*. *cruzi* infections at both the raw protein and phospho-site abundance levels. As expected, we found a predominantly IFN-driven immune response as evidenced by infiltration of immune cells and activation of the JNK and p38 pathways. We also found a downregulation of many mitochondrial proteins which may be linked to the known mitochondrial defects observed in *T*. *cruzi* infections. Our phospho-proteomic analyses revealed potential up- and downstream mediators of JNK, p38 and NDRG2 signaling and identified additional kinase families that may be activated or repressed. Finally, we highlight and discuss the potential repurposing of FDA approved drugs for the reduction of Chagas disease burden. Together, these studies provide a systems-level overview of chagasic hearts and sets the groundwork for future studies to validate the functional consequence of these alterations.

## Methods

### Experimental design and statistical rationale

Multiplexed, quantitative MS experiments were performed in independent, biological duplicate (two mice per condition). *P*-values for altered proteins and phospho-sites were determined by Student’s t-test with Welch’s correction if the variances were unequal. Pi scores[40] corresponding to alpha < 0.05 were considered significant. No adjustment for multiple hypothesis testing was performed. Quantitative data was binned and plotted to ensure normal distributions. Due to the low numbers of replicates assessed, we performed a thorough comparison of our results to previous studies[53–55] (S3 Fig) and validated key findings using western blot.

### Ethics statement

Research utilizing animals adhered to the Animal Welfare Act and Regulations (USDA/APHIS), Public Health Service Policy on Humane Care and Use of Laboratory Animals (OLAW/PHS Policy, AVMA Guidelines for the Euthanasia of Animals: 2013 Edition, and complied to the principles stated in the Guide for the Care and Use of Laboratory Animals, National Research Council, 2011. The facility where this research was conducted is fully accredited by the Association for Assessment and Accreditation of Laboratory Animal Care International. Animal research was conducted under approved protocol S14187 from the Institutional Animal Care and Use Committee, University of California, San Diego.

### Animals

Six week old female C57Bl/6 mice, weighing 16–18 g were used for the animal experiments. Mice were housed and kept in a conventional room at 20 to 24°C under a 12 hour (hr)/12 hr light/dark cycle. The animals were provided with sterilized water and chow ad libitum.

### Chagas disease infection model

*T*. *cruzi* Sylvio X10/4 was maintained in C2C12 myoblasts culture. After 5–7 days of passage of cells and parasites, trypomastigotes released in the supernatant were collected after centrifugation for 15 minutes (min) at 3300 rpm, re-suspended in Dulbecco’s Modified Eagle Medium (DMEM), and used to infect mice by intraperitoneal injection with 1x10^6^ trypomastigote form of *T*. *cruzi*/mouse. The infected groups were age and sex matched with uninfected controls and kept under the same conditions. The general health of the mice was evaluated weekly for one year. At 1-year post infection, the development of heart disease was monitored by electrocardiography at Seaweed Canyon Cardiovascular Physiology Laboratory, Institute for Molecular Medicine, UCSD. After ECG analysis of the mice, heart tissue was collected for further analysis.

### Surface ECG

Adult mice were anesthetized with isoflurane (5% induction, 1–1.5% maintenance in 100% oxygen) and placed on a warming pad (35°C– 37°C). Needle electrodes made of 27-gauge needles were inserted subcutaneously into each of the four limbs and the chest area. Simultaneous standard ECG leads I, II, and chest leads were recorded at a frequency response of 0.05–500 Hz. The signal was digitized and recorded at 2,000 Hz on LabChart (ADinstruments).

### Histology

Upon euthanasia, the hearts were collected from the mice and cut in half in the sagittal orientation, placed in cryomolds, embedded in Tissue-Tek (O.C.T., Sakura Finetek) and snap frozen in liquid nitrogen. Samples were sectioned in a cryostat, fixed in buffered formalin and stained with hematoxylin and eosin or Sirius Red/Fast Green to stain collagen fibers. The slides were scanned using Nanozoomer Slide Scanner (Hamamatsu Photonics, NJ, USA) and images were obtained through NDP viewer software (Hamamatsu Photonics, NJ, USA).

### Histopathology analysis

Levels of inflammation and fibrosis were quantified as previously described[128]. Briefly, 5 random images of mouse hearts (10X magnification) were obtained from each animal, comprising most of the heart section area. Lymphocyte nuclei was segmented through the Particle Analyzer Image processing plugin from FIJI software[129], and lymphocyte nuclei were counted. To measure fibrosis, the red staining of collagen fibers was segmented through color thresholding, and the area fraction of collagen was measured in the generated binary image after segmenting.

### Tissue lysis and protein digestion

Heart tissue was homogenized in a buffer consisting of 3% SDS, 75 mM NaCl, 1 mM β-glycerophosphate, 1 mM NaF, 1 mM Na_3_VO_4_, 10 mM Na_4_P_2_O_7_, 1 mM phenylmethanesulfonyl fluoride and 1X Roche cOmplete mini EDTA free protease inhibitor in 50 mM HEPES, pH 8.5. Homogenization was conducted using a bead beater 3X for 1 min each time with a 1 min rest in between each session. Homogenates were sonicated for 5 min in a water bath sonicator to ensure complete lysis. Lysate supernatants were transferred to a new tube and any remaining cellular debris was pelleted via centrifugation (5 min, 16,000 x *g*, 4°C). The resulting, clarified supernatant was processed for downstream analysis.

Proteins were denatured by addition of an equal volume of 8 M Urea, 50 mM HEPES, pH 8.5. Disulfide bonds were reduced and alkylated by sequential addition of DTT and IAA, respectively[130]. The proteins were then precipitated using methanol and chloroform as previously described[131]. Precipitated proteins were dried on a heat block before being resuspended in 1 M Urea 50 mM HEPES, pH 8.5 for digestion. Protein digestion was performed in a two-step process by digesting with LysC overnight at room temperature (RT) followed by digesting with trypsin for 6 hrs at 37°C. Digested peptides were desalted using C_18_ solid-phase extraction[132]. Desalted peptides were dried in a speed-vac, resuspended in a buffer composed of 50% acetonitrile/5% formic acid and quantified using the Thermo Fisher Colorimetric Peptide Quant Assay according to manufacturer instructions. From each sample, a 50 μg aliquot was taken for standard proteomic analysis while the rest of the sample was saved a phospho-peptide enrichment protocol. Aliquots of peptides were dried in a speed-vac prior to subsequent analyses.

### Enrichment of phospho-peptides

Phospho-peptides were enriched by TiO_2_ beads as previously described[38]. Briefly, the following buffers were made. Binding buffer: 2 M lactic acid, 50% acetonitrile; wash buffer: 50% acetonitrile/0.1% trifluoracetic acid; elution buffer: 50 mM KH_2_PO_4_, pH 10. TiO_2_ beads were washed (once with binding buffer, once with elution buffer and twice with binding buffer). Peptides were resuspended in binding buffer, mixed with beads at a ratio of 1:4 (peptides to beads) and vortexed at RT for 1 hr. Beads were then washed three times with binding buffer, followed by three times with wash buffer. Phospho-peptides were eluted from the beads with elution buffer (two 5 min incubation while vortexing). Enriched peptides were desalted with solid-phase extraction columns then lyophilized and stored at −80°C until they were labeled for quantitation.

### TMT labeling and bRPLC

Samples were labeled with tandem mass tags (TMT) 10-plex reagents for multiplexed, quantitative proteomics[133, 134]. TMT reagents were used to label the digests in random order. Labeling was conducted for 1 hr at RT and was quenched by addition of 9 μl of 5% hydroxylamine. Samples were then acidified by addition of 50 μl of 1% Trifluoroacetic acid (TFA) and pooled. The pooled, multiplexed samples were desalted using C_18_ solid-phase extraction as above.

Fractionation was carried out by basic pH reverse-phase liquid chromatography with fraction combining as previously described[132]. Briefly, samples were solubilized in 110 μl of 5% formic acid/5% acetonitrile and 100 μl was separated on a 4.6 mm x 250 mm C18 column on an Ultimate 3000 HPLC. The resultant 96 fractions were combined into 24 distinct fractions and dried prior to multiplexed LC-MS^3^ analysis.

### LC-MS^2^/MS^3^ analysis

LC-MS^2^/MS^3^ was performed using an Orbitrap Fusion MS equipped with an in-line Easy-nLC 1000 and chilled autosampler. Fraction were resuspended in 5% acetonitrile/5% formic acid and separated on a home-pulled, home-packed column (I.D. 100 μm, O.D. 360 μm) filled with ~0.5 cm of 5 μm C_4_ resin, ~0.5 cm of 3 μm C_18_ resin and ~29 cm of 1.8 μm C_18_ resin for a total length of 30 cm. Peptides were eluted using a gradient of 11 to 30% acetonitrile in 0.125% formic acid over 165 min followed by 15 min of 100% acetonitrile for a total of 180 min analysis time per fraction. The column was heated to 60°C and electrospray was induced by applying 2000 V through a stainless-steel T-junction connecting the column to the LC.

MS^1^ spectra were acquired in data dependent mode with a scan range of 500–1200 m/z and a resolution of 60,000. Automatic gain control (AGC) was set to 2 x 10^5^ with a maximum ion inject time was 100 ms and a lower threshold for ion intensity of 5 x 10^4^. Ions selected for MS^2^ analysis were isolated in the quadrupole at 0.5 Th. Ions were fragmented using collision-induced dissociation (CID) with a normalized collision energy of 30% and were detected in the linear ion trap with a rapid scan rate. AGC was set to 1 x 10^4^ and a maximum inject time of 35 ms. MS^3^ analysis was conducted using the synchronous precursor selection (SPS) option to maximize TMT quantitation sensitivity[135]. Up to 10 MS^2^ ions were simultaneously isolated and fragmented with high energy collision induced dissociation using a normalized energy of 50%. MS^3^ fragment ions were analyzed in the Orbitrap at a resolution of 60,000. The AGC was set to 5 x 10^4^ using a maximum ion injection time of 150 ms. MS^2^ ions 40 m/z below and 15 m/z above the MS^1^ precursor ion were excluded from MS^3^ selection.

### Peptide identification by proteome discoverer

Resultant data files were processed using Proteome Discoverer 2.1. MS^2^ data were queried against the Uniprot mouse database (downloaded: 05/2017–59,010 entries) using the Sequest algorithm[136]. MS^2^ data were also queried against the *T*. *cruzi* proteome derived from a NCBI genome sequence (downloaded: 09/2017–10,809 entries), but, due to minimal spectrum matches, these data were not included in downstream analyses. A decoy search was also conducted with sequences in reversed order[137, 138]. For MS^1^ spectra, a mass tolerance of 50 ppm was used and for MS^2^ spectra a 0.6 Da tolerance was used. Static modifications included TMT 10-plex reagents on lysine and peptide N-termini and carbamidomethylation of cysteines. Variable oxidation of methionine and phosphorylation of serine, threonine and tyrosine residues were also included in the search parameters. The digest specificity was set to fully tryptic with up to two missed cleavages. Data were filtered to a 1% peptide and protein level false discovery rate using the target-decoy strategy[137, 138].

### Data processing and analysis

Reporter ion intensities were extracted from MS^3^ spectra for quantitative analysis[135, 139]. Protein-level quantitation values were calculated by summing signal to noise values for all peptides per protein meeting the specified filters. Data were normalized in a two-step process as previously described[140]. First, the values for each protein were normalized to an average value of all the samples in the experiment. Then, the values were normalized to the median of each reporter ion channel. Phospho-peptides were normalized the same with the following changes. First, phospho-peptides were summed to the unique peptide level rather than protein level. Second, phospho-peptide abundance was normalized to their respective total protein abundance from the standard proteomic experiments. Treatment conditions were compared using an unpaired Student’s t-test with Welch’s correction if the variances were unequal. Pi score[40] was used as a final measure of significance and to rank interesting hits. Gene ontology[42, 43] and functional protein association networks[41] were used to identify enriched groups of related proteins. PTMphinder[60] was used to extract protein locations and flanking motifs and motif-x[62, 141] was used to identify enriched motifs. GPS[59] was used to predict active kinases and their corresponding sites on target proteins.

### Western blot analysis

Western blots were performed on identical samples to the proteomic experiments. Samples were prepared by grinding minced pieces of heart tissue with mortar and pestle using lysis buffer (50 mM Tris, 150 mM NaCl, 1% Triton X-100, pH 8.0) containing 1X Roche cOmplete mini EDTA-free phosphatase inhibitor and protease inhibitor cocktails (2 mM AEBSF, 0.3 μM Aprotinin, 130 μM Bestatin, 1 mM EDTA, 14 μM E-64, and 1 μM Leupeptin—Sigma Chemical Co.). After separating 20 μl for protein quantification, 4X Laemli buffer (Bio-Rad) + 25mM of DTT was added and the samples were boiled at 100 ^o^C for 5 min to guarantee complete inactivation of phosphatases. The protein concentration was determined, and 10 μg or 20 μg of total protein extracts were subjected to electrophoresis on a 12% polyacrylamide gel containing SDS (SDS-PAGE). The electrophoretic separated proteins were transferred to nitrocellulose membranes and incubated for 1 hr at 4°C with blocking buffer consisting of 25 mM Tris, 150 mM NaCl and 0,05% Tween 20 (TBST), 3% non-fat dry milk (Molico) and 0.1% Tween 20. After blocking, the membranes were incubated with anti-IRGM1 (1:200; ABclonal Technology), anti-phosphorylated NDRG2 (1:1000; Signalaway Antibody, recognizes Phospho-T348), anti-total NDRG2 (1:500; ABclonal Technology), anti-phosphorylated p38 (1:200; Santa Cruz Biotechnology, recognizes Phospho-Y182) antibodies for 18 hrs at 4°C. Anti-GAPDH antibodies (1:100; Santa Cruz Biotechnology) were used as internal controls. The membranes were then washed with 1X TBST and incubated for 1 hr at room temperature with anti-rabbit (1:20,000) or anti-mouse (1:30,000) peroxidase conjugated antibodies (Pierce Biotechnology) in blocking buffer. The membranes were washed, and the peroxidase was revealed by chemiluminescence using the Super Signal West Pico (Pierce Biotechnology) kit, imaged by ChemiDoc MP Imaging System (Bio-Rad). Densitometry of the resulting bands was performed with the Image J program (http://rsbweb.nih.gov/ij/).

## Supporting information

S1 Fig Extended Overview of Proteome/Phospho-proteome Results. *A*, Proportion of phospho-peptides with corresponding total protein levels detected in the standard proteomic workflow. *B*, Percent coefficient of variation (% CV) of biological replicates from standard and phospho-proteomic workflows. *C*, Correlation matrix of biological replicates from standard proteomic workflow. *D*, Correlation matrix of biological replicates from phospho-proteomic workflow. *E*, Volcano plot of standard proteins. *F*, Volcano plot of phospho-peptides. For volcano plots, significantly altered features (pi score < 0.05) are highlighted in blue (decreased expression upon infection) or red (increased expression upon infection).
(TIFF)

S2 Fig Concordance of Proteome Findings with Previous Studies. *A*, Significantly altered proteins detected in the standard proteomics experiments known to be altered during Chagas disease. *B*, Immune cell marker proteins detected in standard proteomic experiments. *C*, Proteins that overlap between the standard proteomic experiments and a GWAS of Chagas complications. *D*, Correlation matrix of significantly altered genes in organ-level Chagas infections and genetic cardiomyopathies. *E*, Scatter plot of significantly altered genes in chagasic hearts at the protein and RNA level with pie chart of direction agreements. *F*, Scatter plot of significantly altered genes in chagasic hearts (protein level) and placentas (RNA level) with pie chart of direction agreements. *G*, Scatter plot of significantly altered genes in chagasic hearts (protein level) and DCM hearts (RNA level) with pie chart of direction agreements. *H*, Scatter plot of significantly altered genes in chagasic hearts (protein level) and HF hearts (RNA level) with pie chart of direction agreements. (DCM–dilated cardiomyopathy; HF–heart failure). Significance is noted in reference to the pi score cutoffs for respective significance thresholds (* - α < 0.05; ** - α < 0.01; *** - α < 0.001; **** - α < 0.0001).
(TIFF)

S3 Fig Phospho-protein Families with Numerous Significantly-Altered Phospho-peptides. *A*, Bar graphs of significantly altered SORBS1 phospho-peptides. *B*, Bar graph of total SORBS1 protein. *C*, Bar graphs of significantly altered SORBS2 phospho-peptides. *D*, Bar graph of total SORBS2 protein. *E*, Bar graphs of significantly altered MYH6 phospho-peptides. *F*, Bar graph of total MYH6 protein. *G*, Bar graphs of significantly altered MYBPC3 phospho-peptides. *H*, Bar graph of total MYBPC3 protein. *I*, Bar graphs of significantly altered MYO18A phospho-peptides. *J*, Bar graph of total MYO18A protein. *K*, Bar graphs of significantly altered MYL2 phospho-peptides. *L*, Bar graph of total MYL2 protein. *M*, Bar graphs of significantly altered MYOM1 phospho-peptides. *N*, Bar graph of total MYOM1 protein. *O*, Bar graphs of significantly altered MYOZ2 phospho-peptides. *P*, Bar graph of total MYOZ2 protein. Significance is noted in reference to the pi score cutoffs for respective significance thresholds (* - α < 0.05; ** - α < 0.01; *** - α < 0.001; **** - α < 0.0001).
(TIFF)

S4 Fig Overview of Kinase Prediction Results. *A*, Bar graph depicting number of unique kinases predicted in each kinase group. *B*, Pie chart depicting proportion of predicted target sites for each kinase group. *C*, Proportion of predicted target sites with increased and decreased abundances. *D*, Proportion of predicted target sites with increased and decreased abundances stratified by kinase group. *E*, Proportion of predicted target sites with increased and decreased abundances stratified by kinase family. *F*, Proportion of predicted target sites with increased and decreased abundances stratified by individual kinases. For *C-F* significance was determined by a Chi-squared test (* - α < 0.05; ** - α < 0.01; *** - α < 0.001; **** - α < 0.0001).
(TIFF)

S1 Table Quantified proteins. (XLSX)

S2 Table Quantified phospho-peptides. (XLSX)

S3 Table GO results from standard proteomic experiments. (XLSX)

S4 Table GO results from phospho-proteomic experiments. (XLSX)

S5 Table Kinases predicted from significantly altered phospho-proteomic data. (XLSX)

S6 Table Motif enrichment in significantly altered phospho-proteomic data. (XLSX)

S7 Table Putative kinase-substrate pairs from kinase prediction and motif analysis. (XLSX)

S8 Table Protein-drug interaction pairs from DrugBank. (XLSX)

S9 Table GO results from druggable network. (XLSX)
