# Supplementary figures and images for: Molecular dissection of Chagas induced cardiomyopathy reveals central disease associated and druggable signaling pathways
Source: PLoS Negl Trop Dis. 2020 May 20;14(5):e0007980. doi: 10.1371/journal.pntd.0007980 (PMC7279607; doi:10.1371/journal.pntd.0007980)

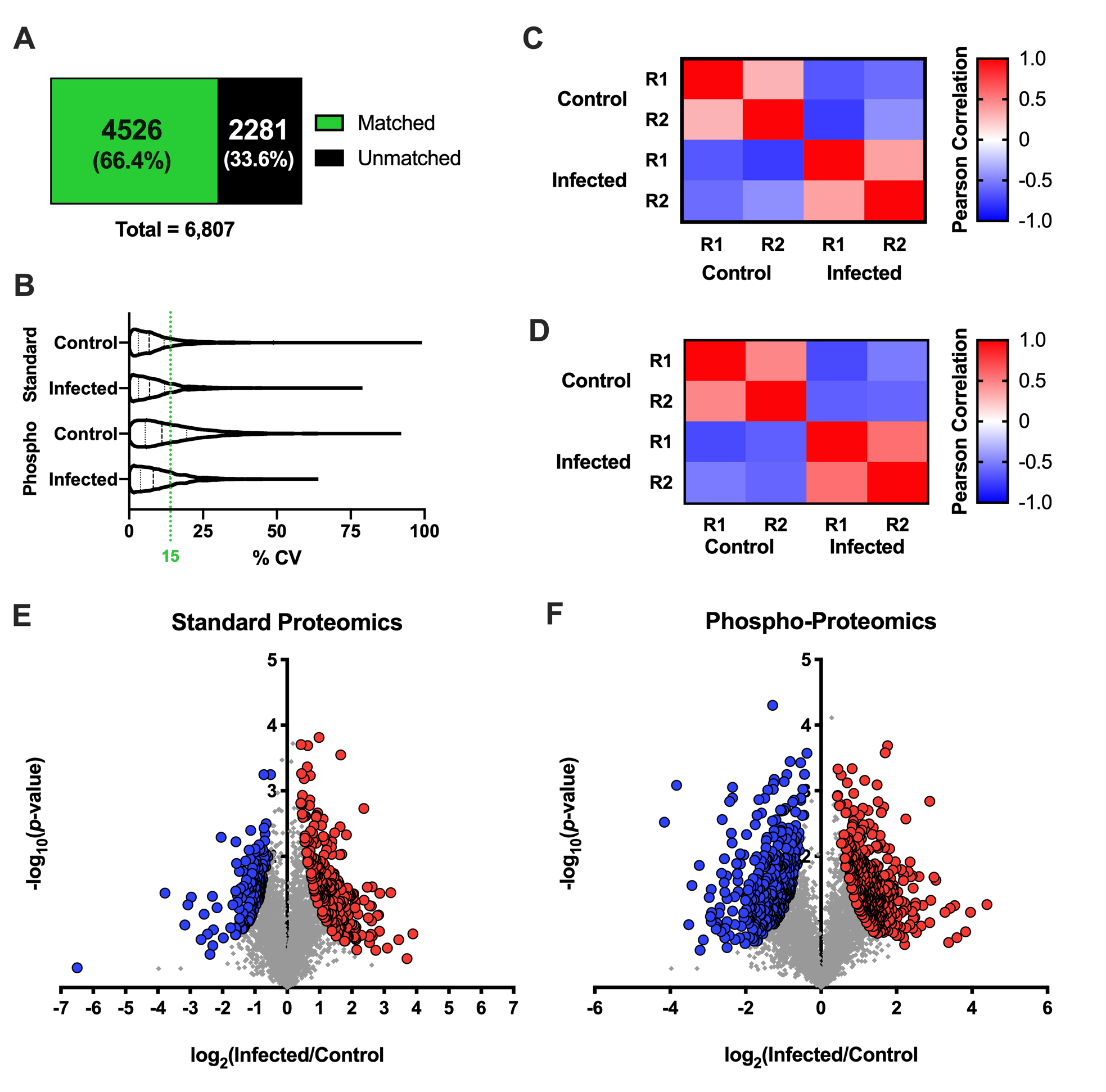

Supplement: S1 Fig — A, Proportion of phospho-peptides with corresponding total protein levels detected in the standard proteomic workflow. B, Percent coefficient of variation (% CV) of biological replicates from standard and phospho-proteomic workflows. C, Correlation matrix of biological replicates from standard proteomic workflow. D, Correlation matrix of biological replicates from phospho-proteomic workflow. E, Volcano plot of standard proteins. F, Volcano plot of phospho-peptides. For volcano plots, significantly altered features (pi score < 0.05) are highlighted in blue (decreased expression upon infection) or red (increased expression upon infection). (TIFF) [file pntd.0007980.s001.tiff]

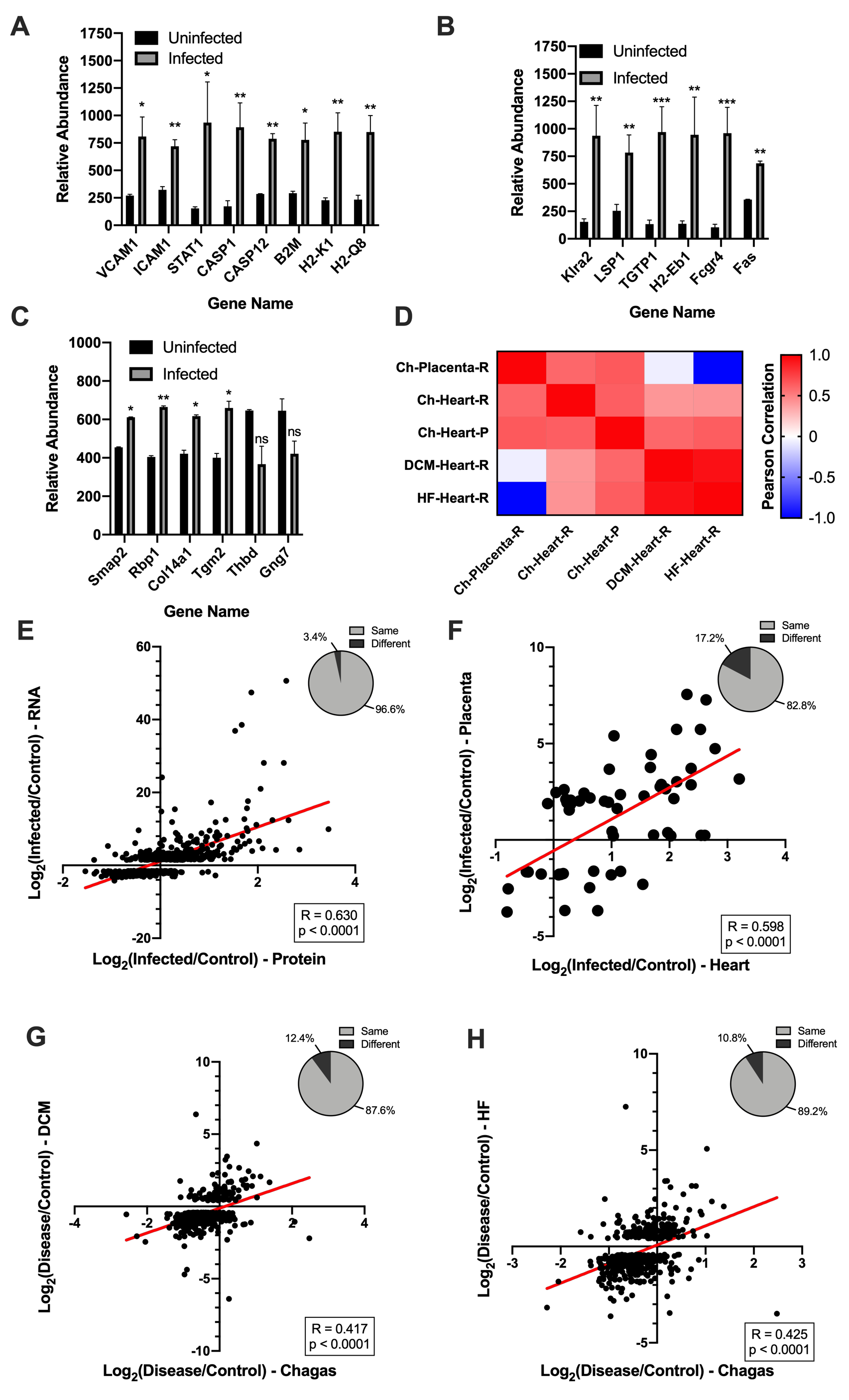

Supplement: S2 Fig — A, Significantly altered proteins detected in the standard proteomics experiments known to be altered during Chagas disease. B, Immune cell marker proteins detected in standard proteomic experiments. C, Proteins that overlap between the standard proteomic experiments and a GWAS of Chagas complications. D, Correlation matrix of significantly altered genes in organ-level Chagas infections and genetic cardiomyopathies. E, Scatter plot of significantly altered genes in chagasic hearts at the protein and RNA level with pie chart of direction agreements. F, Scatter plot of significantly altered genes in chagasic hearts (protein level) and placentas (RNA level) with pie chart of direction agreements. G, Scatter plot of significantly altered genes in chagasic hearts (protein level) and DCM hearts (RNA level) with pie chart of direction agreements. H, Scatter plot of significantly altered genes in chagasic hearts (protein level) and HF hearts (RNA level) with pie chart of direction agreements. (DCM–dilated cardiomyopathy; HF–heart failure). Significance is noted in reference to the pi score cutoffs for respective significance thresholds (* - α < 0.05; ** - α < 0.01; *** - α < 0.001; **** - α < 0.0001). (TIFF) [file pntd.0007980.s002.tiff]

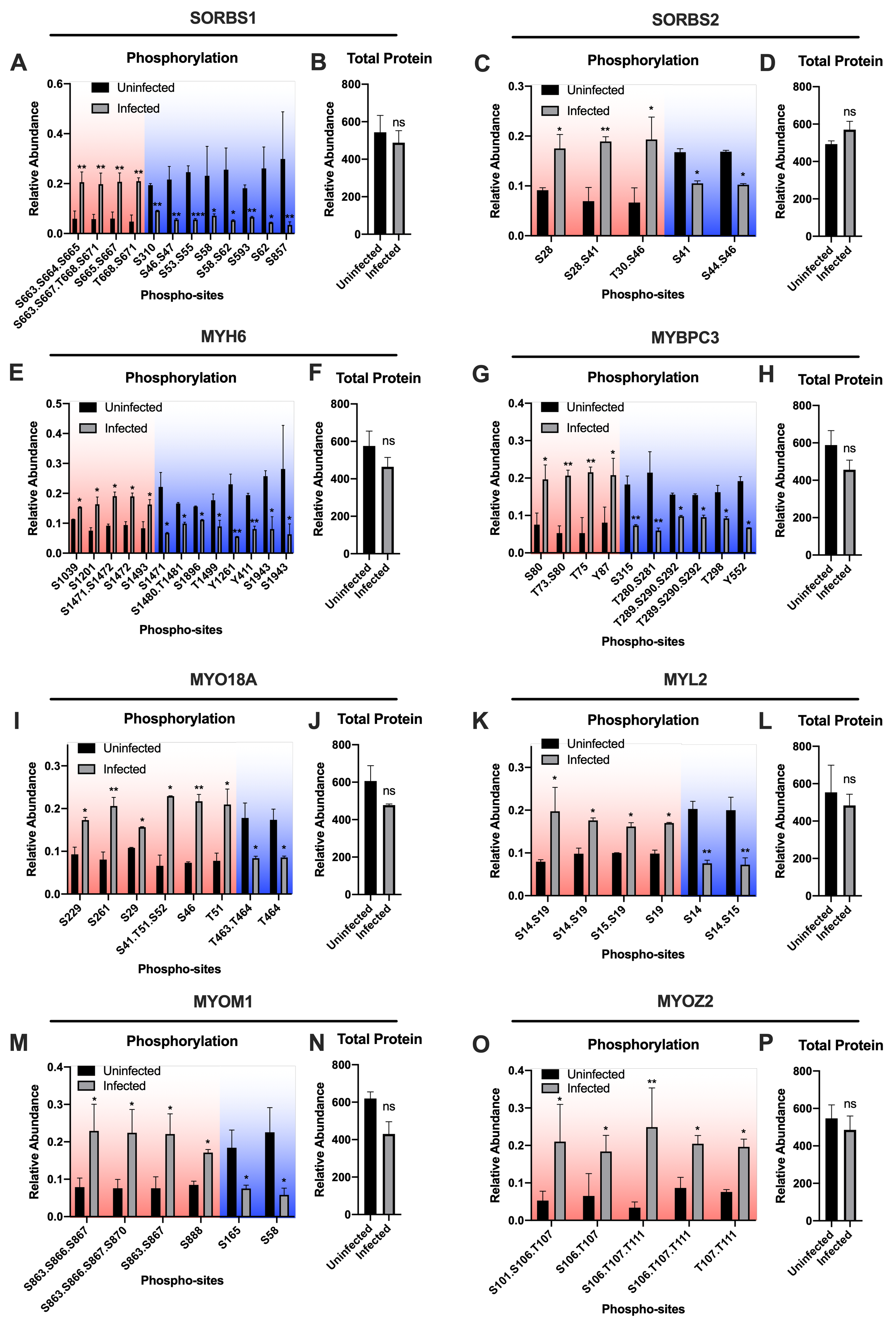

Supplement: S3 Fig — A, Bar graphs of significantly altered SORBS1 phospho-peptides. B, Bar graph of total SORBS1 protein. C, Bar graphs of significantly altered SORBS2 phospho-peptides. D, Bar graph of total SORBS2 protein. E, Bar graphs of significantly altered MYH6 phospho-peptides. F, Bar graph of total MYH6 protein. G, Bar graphs of significantly altered MYBPC3 phospho-peptides. H, Bar graph of total MYBPC3 protein. I, Bar graphs of significantly altered MYO18A phospho-peptides. J, Bar graph of total MYO18A protein. K, Bar graphs of significantly altered MYL2 phospho-peptides. L, Bar graph of total MYL2 protein. M, Bar graphs of significantly altered MYOM1 phospho-peptides. N, Bar graph of total MYOM1 protein. O, Bar graphs of significantly altered MYOZ2 phospho-peptides. P, Bar graph of total MYOZ2 protein. Significance is noted in reference to the pi score cutoffs for respective significance thresholds (* - α < 0.05; ** - α < 0.01; *** - α < 0.001; **** - α < 0.0001). (TIFF) [file pntd.0007980.s003.tiff]

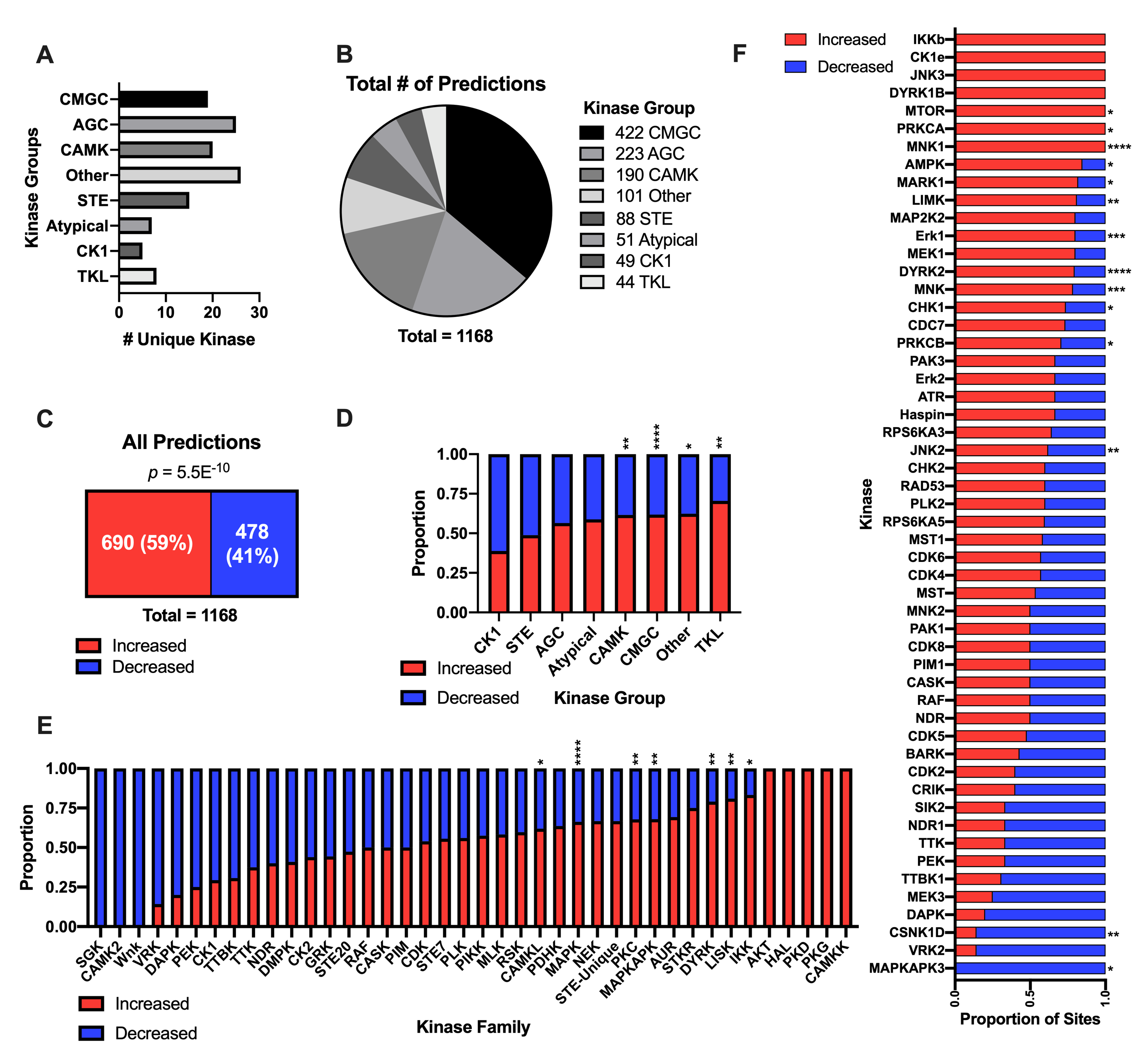

Supplement: S4 Fig — A, Bar graph depicting number of unique kinases predicted in each kinase group. B, Pie chart depicting proportion of predicted target sites for each kinase group. C, Proportion of predicted target sites with increased and decreased abundances. D, Proportion of predicted target sites with increased and decreased abundances stratified by kinase group. E, Proportion of predicted target sites with increased and decreased abundances stratified by kinase family. F, Proportion of predicted target sites with increased and decreased abundances stratified by individual kinases. For C-F significance was determined by a Chi-squared test (* - α < 0.05; ** - α < 0.01; *** - α < 0.001; **** - α < 0.0001). (TIFF) [file pntd.0007980.s004.tiff]
